# Towards Computer-Based Automated Screening of Dementia Through Spontaneous Speech

**DOI:** 10.3389/fpsyg.2020.623237

**Published:** 2021-02-12

**Authors:** Karol Chlasta, Krzysztof Wołk

**Affiliations:** ^1^Department of Computer Science, Polish-Japanese Academy of Information Technology, Warsaw, Poland; ^2^Institute of Psychology, SWPS University of Social Sciences and Humanities, Warsaw, Poland

**Keywords:** dementia detection, prosodic analysis, affective computing, transfer learning, convolutional neural network, machine learning, speech technology, mental health monitoring

## Abstract

Dementia, a prevalent disorder of the brain, has negative effects on individuals and society. This paper concerns using Spontaneous Speech (ADReSS) Challenge of Interspeech 2020 to classify Alzheimer's dementia. We used (1) VGGish, a deep, pretrained, Tensorflow model as an audio feature extractor, and Scikit-learn classifiers to detect signs of dementia in speech. Three classifiers (LinearSVM, Perceptron, 1NN) were 59.1% accurate, which was 3% above the best-performing baseline models trained on the acoustic features used in the challenge. We also proposed (2) DemCNN, a new PyTorch raw waveform-based convolutional neural network model that was 63.6% accurate, 7% more accurate then the best-performing baseline linear discriminant analysis model. We discovered that audio transfer learning with a pretrained VGGish feature extractor performs better than the baseline approach using automatically extracted acoustic features. Our DepCNN exhibits good generalization capabilities. Both methods presented in this paper offer progress toward new, innovative, and more effective computer-based screening of dementia through spontaneous speech.

## 1. Introduction

One of the most important social problems in developed countries is the constant rise of the percentage of the elderly population. A major health issue affecting this segment of population is the appearance Alzheimer's dementia (AD), affecting around 50 million people worldwide and expected to grow three times over the next 50 years (Baldas et al., 2010).

Dementia is estimated to be responsible for 11.2% of years lived with disability in people over 60 years of age, compared with 9.5% for stroke, 5.0% for cardiovascular disease, and 2.4% for cancer. In Europe, the prevalence of AD increases exponentially with age. The incidence also increases with age, although with a plateau in extreme old age (Todd and Passmore, 2009).

Comorbidity of several physical and mental health disorders was studied in relation to age and socioeconomic deprivation. The presence of mental health disorders increased as the number of physical morbidities increased, and was much greater in more deprived than in less deprived people. Physical-mental health comorbidity is very common, with depression and painful disorders as key comorbidities, and with dementia seen in a small reverse gradient (Barnett et al., 2012).

There is a significant relation between old-age depression and subsequent dementia in patients over the age of 50. This supports the hypothesis of old-age depression being a predictor, and possibly a causal factor of subsequent dementia (Buntinx et al., 1996).

Speech is a well-established early indicator of cognitive deficits including dementia (Bucks et al., 2000). Speech processing methods offer great potential to fully automatically screen for prototypic indicators in near real time, and they can be used as an additional information source when diagnosing Alzheimer's disease (Weiner et al., 2016).

Dementia was detected in speech with voice activity detection and speaker diarization followed by extraction of acoustic features. The unsupervised system achieved up to 0.645 unweighted average recall (UAR). Authors detected dementia using speech segments as short as 2.5 min, but achieved the best results using segments in the range between 10 and 15 min (Weiner et al., 2018).

Other AD detection approaches combined extraction of acoustic and linguistic features (Speech to Text and Human Transcriptionist), and applied a one-way ANOVA for feature selection. The reported binary classification accuracy on brief (less than 10 min) spontaneous speech samples reached 88%, with recall of 0.920 (Jarrold et al., 2014).

We target the classification task of AD Recognition through Spontaneous Speech (ADReSS Challenge 2020). The AD classification task consists of creating binary classification models to distinguish between AD and non-AD patient speech on the ADReSS dataset. The authors of that challenge prepared the dataset and provided five baseline, machine learning classification models, that used both acoustic and linguistic features for the detection of AD in spontaneous speech. Their acoustic approaches were based on emobase (Eyben et al., 2010), ComParE 2013 (Eyben et al., 2013), Multi-resolution Cochleagram features (MRCG) proposed by Chen et al. (2014), the Geneva minimalistic acoustic parameter set (eGeMAPS) by Eyben et al. (2015), and minimal feature set (Luz, 2017). The best baseline accuracy was achieved by linear discriminant analysis (LDA) model using ComParE features.

In this paper, we propose two methods for speech-based screening of AD. Our models perform significantly better than the ADReSS challenge baseline for classification task, as evaluated on the same, official ADReSS challenge dataset.

## 2. Methods

### 2.1. Dataset

The dataset for the 2020 ADReSS challenge consists of speech recordings elicited for the Cookie Theft picture description task from the Boston Diagnostic Aphasia Exam (Goodglass et al., 2001). These data were balanced by the organizers in terms of age, gender, and the distribution of labels between the training and test partitions in order to minimize the risk of bias in the prediction tasks. The dataset from 78 non-AD subjects, and 78 AD subjects, was labeled for binary classification and regression tasks. The labels for the binary classification include Alzheimer's dementia and healthy control, whereas the labels for the regression task are Mini-Mental State Examination (MMSE) scores (Folstein et al., 1975), which provide a means for dementia diagnosis based on linguistic tests. For more details regarding the dataset, including the segmentation and voice activity detection algorithm, we refer the reader to the ADReSS challenge baseline paper (Luz et al., 2020).

### 2.2. VGGish Model and Scikit-Learn Classifiers

**Figure 1:**
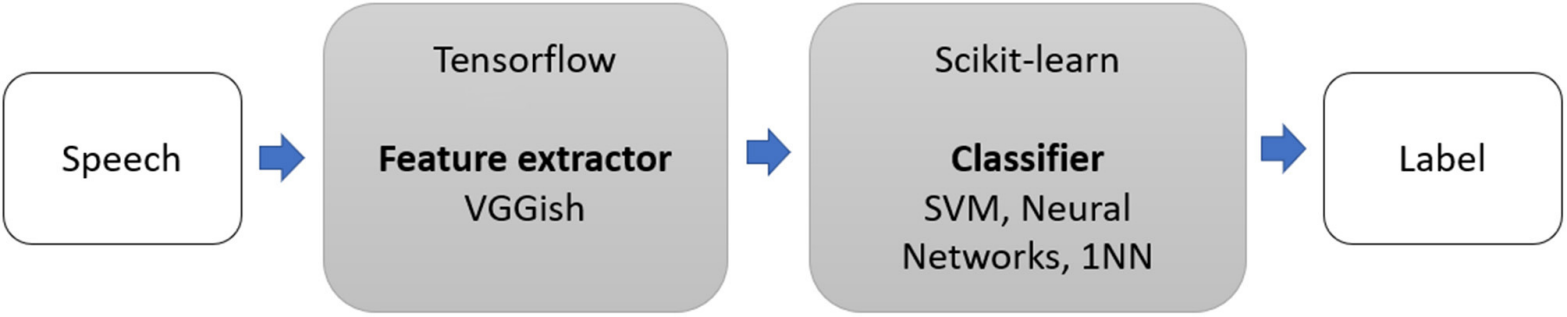
Two-stage architecture: VGGish model and Scikit-learn classifiers.

We extended the method of Pons Puig et al. (2018) and conducted two-step classification experiments to detect cognitive impairment due to AD (as shown in Figure 1). This consisted of a two-stage classification process, where a classifier was trained with features to predict whether a speech segment was uttered by a non-AD or AD patient, and majority vote (MV) classification, which assigned each subject an AD or non-AD label based on the majority labels classification.

#### 2.2.1. Feature Extraction

We used VGGish (Hershey et al., 2017), a deep, pretrained Tensorflow (Abadi et al., 2016) model as a feature extractor. VGGish is an audio embedding produced by training a modified VGGNet model (Simonyan and Zisserman, 2014) to predict video tags from the Youtube-8M dataset (Abu-El-Haija et al., 2016). Principal component analysis (PCA) (Cao et al., 2003) was used for dimensionality reduction, with PCA set to 128. VGGish model converted audio input features into high-level 128-D embedding, which was fed as an input to a downstream classification model. The features were extracted from non-overlapping audio patches of 0.96 s, where each audio patch covered 64 mel bands and 96 frames of 10 ms each.

#### 2.2.2. Classification Methods

We performed classification experiments using five different methods, namely support vector machines (SVM, with a radial basis function kernel and scaling gamm), linear support vector machines (LSVM), perceptron, multi-layer perceptron classifier (MLP, with 20 hidden layers, using a stochastic gradient descent solver, 600 iterations, learning rate of 0.001), and nearest neighbor (1NN, for KNN with *K* = 1 and cosine metric).

### 2.3. DemCNN—Custom Convolutional Neural Network

Current deep convolutional neural network (CNN) performs considerably better than the previous state-of-the-art (Krizhevsky et al., 2012). Transfer learning was often used in medical image analysis (Cheplygina et al., 2019). Applying transfer learning on a wide range of tasks nearly always gave better results (Kornblith et al., 2019). CNN-based methods have been successfully employed to medical imaging tasks and achieved human-level performance in classification tasks (Esteva et al., 2019). CNNs have proven very effective in image classification and show promise for audio (Hershey et al., 2017). We extend the audio classification work presented in Wołk, K., and Wołk (2019) and Chlasta et al. (2019).

#### 2.3.1. Classification Method

**Figure 2:**
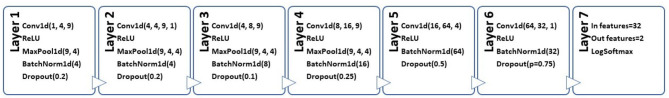
Architecture diagram of DemCNN, a custom PyTorch convolutional neural network for speech classification.

We introduce DemCNN, a custom PyTorch (Paszke et al., 2019) CNN. We designed and implemented a custom sequential architecture consisting of six Conv1D layers using ReLU activation function, batch normalization and dropout, with the final (seventh) output layer being a dense layer. The output layer had 2 nodes (num_labels), which matched the number of possible classifications outputs. Figure 2 presents a more detailed architecture diagram of our custom CNN for speech classification.

We unpacked a byte-string for each file into a 1D numpy array of numbers that could be analyzed by the CNN. Subsequently, the dataset was downsampled with a low-pass filter (with downsampling factors of 4, 4, 2).

We performed a two-step training of our CNN model using a cross-entropy loss function. We fine-tuned learning rate, the number of training cycles, and the number of training iterations per cycle. We set the first (training) batch size to 32, and the second (deployment) batch size to 2. The selection of the second learning rate for each step of our method was automated using a custom function operating on standard lr_finder. We trained the classifier for 2 or 4 epochs.

## 3. Experiments and Results

All experiments were implemented in Python using Scikit-learn (Pedregosa et al., 2011), Tensorflow (Abadi et al., 2016), and PyTorch (Paszke et al., 2019) on the Google Colaboratory Platform (Bisong, 2019). The platform uses Jupyter Notebook standard that facilitates exchange of source code and reproducibility of results. The source code and accompanying results are available on GitHub.^1^

The ADReSS development data were split into train and test sets by randomly assigning 80% of the speakers to the train set and 20% to the test set. Results obtained for different classifier setups are summarized in Table 1.

**Table 1:** Summary of classification results on AD Recognition through Spontaneous Speech (ADReSS) training set.

**Model type**	**Precision**	**Recall**	**F1 score**	**Accuracy**	**Baseline accuracy**
SVM	0.556	0.454	0.500	0.545	0.565 (SVM + Minimal)
LinearSVM	0.600	0.545	0.571	**0.591**	0.565 (SVM + Minimal)
Perceptron	0.600	0.545	0.571	**0.591**	0.565 (LDA + ComParE)
MLP	0.429	0.273	0.333	0.454	0.565 (LDA + ComParE)
1NN	0.600	0.545	0.571	**0.591**	0.574 (1NN + ComParE)
DemCNN	0.692	0.692	0.692	**0.636**	0.565 (LDA + ComParE)

*Our approaches (VGGish + 128 Principal component analysis [PCA] and custom audio convolutional neural network [DemCNN]) vs. the best baseline accuracy on acoustic features. The bold values indicate best results achieved on ADReSS training dataset*.

Three models we developed using the first approach (VGGish + 128 PCA + linearSVM/perceptron/1NN) achieved 59% accuracy in our test set. Employing the same setup with SVN model, we achieved 55% accuracy. The best-performing baseline SVM models using MRCG features proposed by (Chen et al., 2014) and the ComParE 2013 features (Eyben et al., 2013) achieved lower accuracy of 53%. Interestingly, our 1NN model achieved better results than the best-performing baseline 1NN model using ComParE features (59% against 57%).

Our custom raw waveform DemCNN system achieved the best classification accuracy of 63.6%. The model classified 14 speakers correctly, eight incorrectly, and proved the most effective in distinguishing between AD and non-AD speech samples on the full wave enhanced ADReSS audio dataset. This result was 7% better then the best baseline classification accuracy on the ADReSS training set (Luz et al., 2020).

The final results for our custom audio DemCNN model were submitted to the 2020 ADReSS Challenge organizers after retraining the classifier on the full ADReSS training set, and predicting on the full ADReSS test set (see Table 2 for results and the accompanying hyperparameters). Our model performed slightly better (1%) on the test partition than the best baseline LDA model trained on automatically extracted ComParE feature set (Eyben et al., 2013).

**Table 2:** The results of Alzheimer's dementia (AD) classification task on AD Recognition through Spontaneous Speech (ADReSS) test set.

**Approach**	**Class**	**Precision**	**Recall**	**F1 Score**	**Accuracy**
DemCNN (Learning rate = 0.2;	Non-AD	0.528	0.792	0.633	0.542
Cycles = 4.4; Lengths = 8.8)	AD	0.528	0.792	0.389	0.542
DemCNN (Learning rate = 0.1;	Non-AD	0.625	0.625	0.625	**0.625**
Cycles = 2.2; Lengths = 8.8)	AD	0.625	0.625	0.625	**0.625**
Baseline acoustic features	Non-AD	0.670	0.500	0.570	0.620
(LDA + ComParE)	AD	0.600	0.750	0.670	0.620

*Our approach (custom audio convolutional neural network [DemCNN]) vs. the best baseline on acoustic features (linear discriminant analysis [LDA] + CompParE). The bold values indicate best results achieved on ADReSS test dataset*.

## 4. Discussion

The main limitations of the AD field are poor standardization, limited comparability of results, and a degree of disconnect between study aims and clinical applications (de la Fuente Garcia et al., 2020). Our two methods are attempting to close some of these gaps.

Data scarcity has hindered research into the relationship between speech and dementia. Recently, the community has turned to transfer learning (Yosinski et al., 2014), as a solution for a wide range of machine learning tasks for which labeled data are scarce. Selecting the right pretrained model as audio feature extractor allows to rapidly prototype competent speech classifiers.

In our first approach, we used a standard VGGish (Hershey et al., 2017), that is a popular deep audio embedding model trained on Youtube-8M video dataset (Abu-El-Haija et al., 2016). In our experiments to detect subtle changes in pathological speech, we confirmed that automatic extraction of acoustic features (Eyben et al., 2010) performs similarly to using a pretrained deep audio embedding model for feature extraction.

Similarly to us, Syed et al. (2020) also used VGGish deep acoustic embeddings in the ADReSS Challenge. They used other types of feature aggregation methods: (a) Fisher Vector encodings (FVs) and (b) Bag-of-Audio-Words (BoAW). Both achieved satisfactory results. Their VGGish and FVs model overperformed ours (59.1%) with 62.96% accuracy on the train partition, whereas their VGGish and BoAW model achieved even higher accuracy of 75%.

Our second method, the DemCNN model, for which we only performed a basic hyperparameter tuning, improved the classification results further. Moreover, the results achieved by DemCNN were similar in training and testing (63.6 vs. 62.5%), which is a good indicator of the lack of overfitting during the training process. This can be explained by a larger dropout defined in layers 5 and 6 of the network. An expected consequence of that is a good generalization capacity of our DemCNN model, which would positively impact the overall performance in clinical practice, when working with new data.

A similar approach to our DemCNN in the ADReSS Challenge was proposed by Cummins et al. (2020). Their raw segment based End-to-End CNN had four convolution layers, with the first convolution layer used to model voice source-related information or vocal tract information, such as formants. This approach achieved 71.3% accuracy on the training partition, but the reported result on the test partition was only 66.7%. Although this result is 4% better than our DemCNN, an expected consequence of a large difference between the results in training and test partitions is possibly a worse generalization capability of the network when working with new data.

An interesting opportunity for future research would be to use a combination of acoustic and linguistic features in detecting dementia. The latter approach, derived from automatic speech recognition (ASR) output, or from manual transcripts, had already been proven to detect dementia (Weiner et al., 2017), but relatively small gains were found when fusing acoustics and linguistics approaches (Cummins et al., 2020; Rohanian et al., 2020).

ADReSS Challenge 2020 helped to establish that although the linguistic systems outperforms the acoustic systems in AD (Cummins et al., 2020; Yuan et al., 2020), this result is unsurprising given that a human observer generated the transcripts manually, and they contain considerably fewer sources of noise than the audio recordings. As a result, such systems would be difficult to implement in clinical practice.

An option to overcome that would be to combine acoustic information with linguistics systems based on transcripts generated from ASR systems. This idea would introduce automation, but also increase the complexity, and dependency on errors rate for ASR in a given language.

It may also be useful for future work to gather a large dataset combining spontaneous speech samples for several pathologies (starting with depression and dementia, especially for old-age patients) to train an improved DepCNN to distinguish different types of disorders in pathological speech.

Finally, the DementiaBank's Pitt corpus (Jost and Grossberg, 1995) is large enough for considering experiments with other, custom, or off-the-shelf deep neural network architectures.

## 5. Conclusion

In this paper, we proposed and compared two acoustic-based systems: VGGish, a pretrained Tensorflow model as audio feature extractor and Scikit-learn classifiers with DemCNN, a custom raw waveform based CNN.

In the first approach, we selected the VGGish model as feature extractor and PCA for dimensionality reduction. This approach achieved the accuracy of 59.1%, 3% better than the best baseline accuracy achieved on the train partition with acoustic feature extraction for the respective classification algorithms.

In the second approach, we presented DemCNN, our custom PyTorch audio CNN to detect signs of dementia in spoken language. According to the experiments, the proposed architecture achieved promising performance and demonstrated the effectiveness of our method, as well as good generalization capabilities. DemCNN overperformed the best baseline accuracy of LDA model (ComParE feature set) by 7% on the ADReSS training set (accuracy of 63.6%), and 1% on the test ADReSS test set (accuracy of 62.5%). Our DemCNN and End-to-End Convolutional Neural Network (Cummins et al., 2020) produced the strongest performance of the acoustic systems on the ADReSS 2020 classification task, highlighting the benefits of self-learning features.

To conclude, we demonstrated a proof-of-concept, and applicability of (1) audio transfer learning for feature extraction, (2) DemCNN, a custom raw waveform based CNN in detecting dementia through spontaneous speech. We demonstrated that (1) audio transfer learning with a pretrained VGGish feature extractor performs better then the baseline approach (Luz et al., 2020) using automatically extracted acoustic features, and that these are relatively minor improvements. Our DemCNN method (2) overperforms our VGGish method (1) by 4% and the baseline on the test partition (Luz et al., 2020) by roughly 1%.

Both approaches presented are active attempts to close the gaps in standarization of automatic AD detection, and to improve the overall comparability of results to better embed computational speech technology into clinical practice. They offer simplicity, easy deployment, and they are language independent, which could result in a wide adoption and improved accessibility in a short space of time.

This contribution is especially important now, in the time of current COVID-19 pandemic, when the need for a remote digital health assessment tool is greater than ever for the elderly and other vulnerable populations.

## Data Availability Statement

Publicly available datasets were analyzed in this study. This data can be found here: https://dementia.talkbank.org/.

## Author Contributions

KC was responsible for conceptualization, algorithmic development, data analysis, investigation, validation, and writing of original draft. KW supervises the entire work and contributes to idea conceptualization, algorithmic development, manuscript revision, and approval of the submission.

## Conflict of Interest

The authors declare that the research was conducted in the absence of any commercial or financial relationships that could be construed as a potential conflict of interest.

## Footnotes

^1^Code: https://github.com/KarolChlasta/ADReSS-Challenge2020
